# The genome sequence of Ashworth’s Rustic, *Xestia ashworthii* (Doubleday, 1855)

**DOI:** 10.12688/wellcomeopenres.20499.1

**Published:** 2023-12-20

**Authors:** John F. Mulley, David C. Lees, Natural History Museum Genome Acquisition Lab, Darwin Tree of Life Barcoding collective, Wellcome Sanger Institute Tree of Life Management, Samples and Laboratory team, Wellcome Sanger Institute Scientific Operations: Sequencing Operations, Wellcome Sanger Institute Tree of Life Core Informatics team, Tree of Life Core Informatics collective, Darwin Tree of Life Consortium

**Affiliations:** 1 School of Natural Sciences, Bangor University, Bangor, Wales, UK; 2 Natural History Museum, London, England, UK

**Keywords:** Xestia ashworthii, Ashworth’s Rustic, genome sequence, chromosomal, Lepidoptera

## Abstract

We present a genome assembly from an individual male *Xestia ashworthii* (Ashworth’s Rustic; Arthropoda; Insecta; Lepidoptera; Noctuidae). The genome sequence is 726.3 megabases in span. Most of the assembly is scaffolded into 31 chromosomal pseudomolecules, including the z sex chromosome. The mitochondrial genome has also been assembled and is 15.39 kilobases in length.

## Species taxonomy

Eukaryota; Metazoa; Eumetazoa; Bilateria; Protostomia; Ecdysozoa; Panarthropoda; Arthropoda; Mandibulata; Pancrustacea; Hexapoda; Insecta; Dicondylia; Pterygota; Neoptera; Endopterygota; Amphiesmenoptera; Lepidoptera; Glossata; Neolepidoptera; Heteroneura; Ditrysia; Obtectomera; Noctuoidea; Noctuidae; Noctuinae; Noctuini; *Xestia*; *Xestia ashworthii* ( Doubleday, 1855) (NCBI:txid988043).

## Background

Ashworth’s Rustic, *Xestia ashworthii* ( Doubleday, 1855) is a medium-sized (16–20 mm forewing length: Waring *et al.*, 2017) noctuid, that Henry Doubleday based on a specimen collected at Llangollen in Denbighshire in 1853 by Joseph Ashworth. In the UK at least, the forewing ground colour is ashy to dark grey with darker grey cross markings, the post reniform one narrow and very jagged.

Ashworth’s Rustic is found only in Wales in the UK, and more specifically areas of north and west Wales ( NBN Atlas Partnership, 2023; Randle *et al.*, 2019). It was found in Anglesey in 1994 ( Waring *et al.*, 2017). In Europe also, it is associated with mountainous regions and areas of exposed rock and scree slopes, ranging from the Pyrenees, Eastern Europe, Southern Scandinavia, and sparsely distributed from Italy, Greece, and eastwards in Asia to Lake Baikal ( GBIF Secretariat, 2023).

In Wales there are relatively few records of Ashworth’s Rustic annually due to low sampling intensity. However, where concerted efforts have been made, the species has been found to be locally abundant ( Morgan, 1974). Randle *et al.* (2019) were unable to characterise distribution and abundance changes due to under-recording. The species was classified as nationally rare in 2019 review of the status of the macro-moths of Great Britain ( Fox *et al.*, 2019) or Nationally Scarce A ( Waring *et al.*, 2017). It is also a priority species on the UK Biodiversity Action Plan ( BRIG, 2007).

Biological details owe much to Tait (1923), Crewdson (1964), and Morgan (1974). Adults fly in the UK from end of June and into early August with a peak in mid-July ( Randle *et al.*, 2019).

Eggs are initially white and then turn brown over a few days, and are laid in batches on various plants, including grass stems and exposed heather roots. Heath Bedstraw ( *Galium saxatile*) and heathers seem to be the most important foodplants ( Bretherton *et al.*, 1983: 182 characterise them as polyphagous). The looping small larvae overwinter from autumn, and can be found feeding again from February onwards, with large caterpillars, 4 cm fully fed, grey, with a large wedge-shaped black marking bilaterally on each segment, often found in April and May apparently basking on exposed rocks or near the top of vegetation. Pupae have been recovered from cracks and crevices in rocks, and from shallow chambers under the soil, where they form a flimsy cocoon ( Bretherton *et al.*, 1983: 182). 

Tait (1923) reported that the previously easily-accessible and apparently healthy populations around Penmaenmawr and the Sychnant Pass suffered greatly from attack by a still unidentified ichneumonid wasp around 1917–18. A nationally notable tachinid fly, *Drino lota* (Meigen, 1824) has been reported as a parasitoid of *X. ashworthii* ( Smith, 1989: 124).

The continental populations have been classified into a number of subspecies, including *Xestia ashworthii artvina* (de Freina & Hacker, 1985); *Xestia a. candelarum* (Staudinger, 1871); *Xestia a. centralaltaica* (Volynkin & Dvorak, 2016); *Xestia a. jotunensis* (Schoyen, 1887); and *Xestia a. lactescens* (Turati, 1919). The Welsh subspecies of Ashworth’s Rustic ( *Xestia ashworthii ashworthii*) most likely represents a glacial relic ( Jiménez-Alfaro *et al.*, 2016). It belongs to the cluster on BOLD (BIN), BOLD:ABY8432. The mitochondrial genome sequence (OX465578.1, for NHMUK014425722) is, at least, one nucleotide divergent (0.15% for 658 bp) to most specimens of the last cluster, a few of which identified as *X. x. candelarum*, A DNA barcode from the same specimen (BOLD Process ID DTNHM5433-23) conflicts by one nucleotide (is at least 0.31% divergent to others). The mitochondrial genome sequence for NHMUK014425722 is most closely related (at 1.98-2.16% divergence for 658 bp) to *Xestia wockei* (Möschler, 1862) from Russia and *X. scropulana* (Morrison, 1874) in Canada both species belonging to BIN BOLD:ABY8431, and the latter taxon possibly representing a synonym of the former.

The recent finding that cool-adapted moths in Great Britain are increasingly shifting their range to the north-west ( Hordley *et al.*, 2023) raises significant issues for Ashworth’s Rustics, as there is limited habitat available to expand into in north Wales before they meet the Irish Sea, and even elevational shifts as seen in mountain butterflies ( Rödder *et al.*, 2021) can only occur for so long before they reach the peak.

This genome sequence will not only be an invaluable resource for determination of levels of genetic differentiation within and between the subspecies, and their relationships, also to other *Xestia*, but will be of great help in developing genetic markers to assess levels of genetic diversity and patterns of gene flow within the Welsh and continental populations, and for detecting differential patterns of selection and evolutionary trajectories across their range.

## Genome sequence report

**Figure 1.**
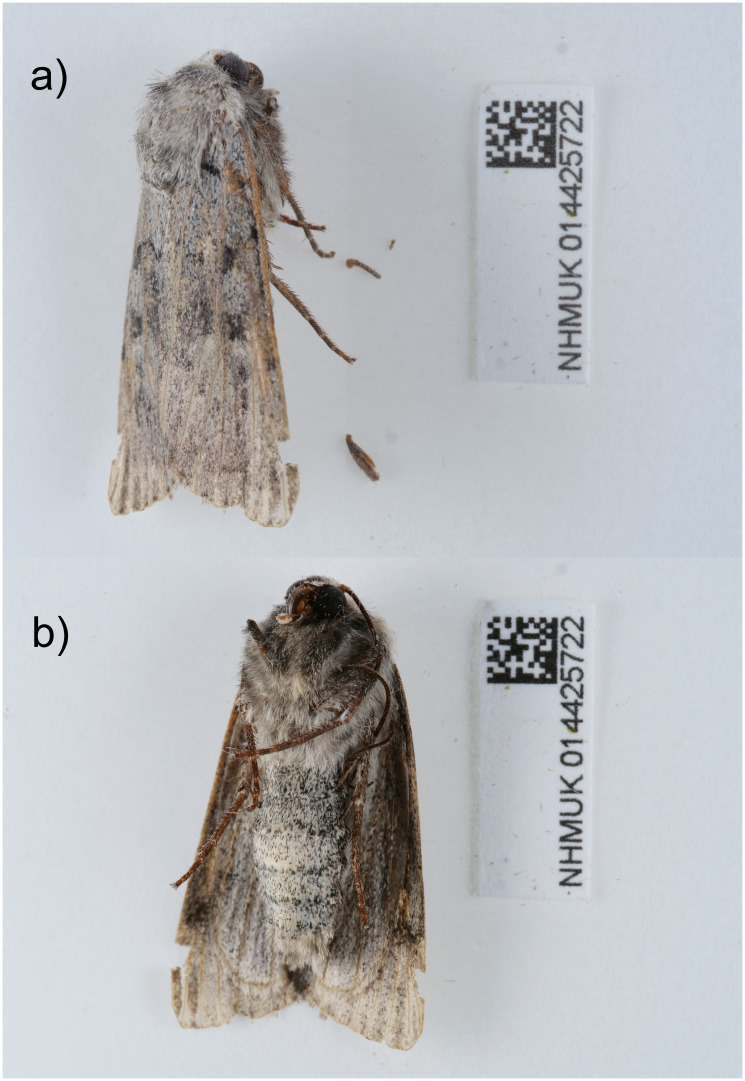
Photograph of the *Xestia ashworthii* (ilXesAshw1) specimen used for genome sequencing.

The genome was sequenced from one male *Xestia ashworthii* ( Figure 1) collected from Llanfihangel-y-Pennant, Wales (52.67, –3.96). A total of 29-fold coverage in Pacific Biosciences single-molecule HiFi long reads was generated. Primary assembly contigs were scaffolded with chromosome conformation Hi-C data. Manual assembly curation corrected 5 missing joins or mis-joins and removed 7 haplotypic duplications, reducing the assembly length by 0.42% and the scaffold number by 6.67%.

**Figure 2.**
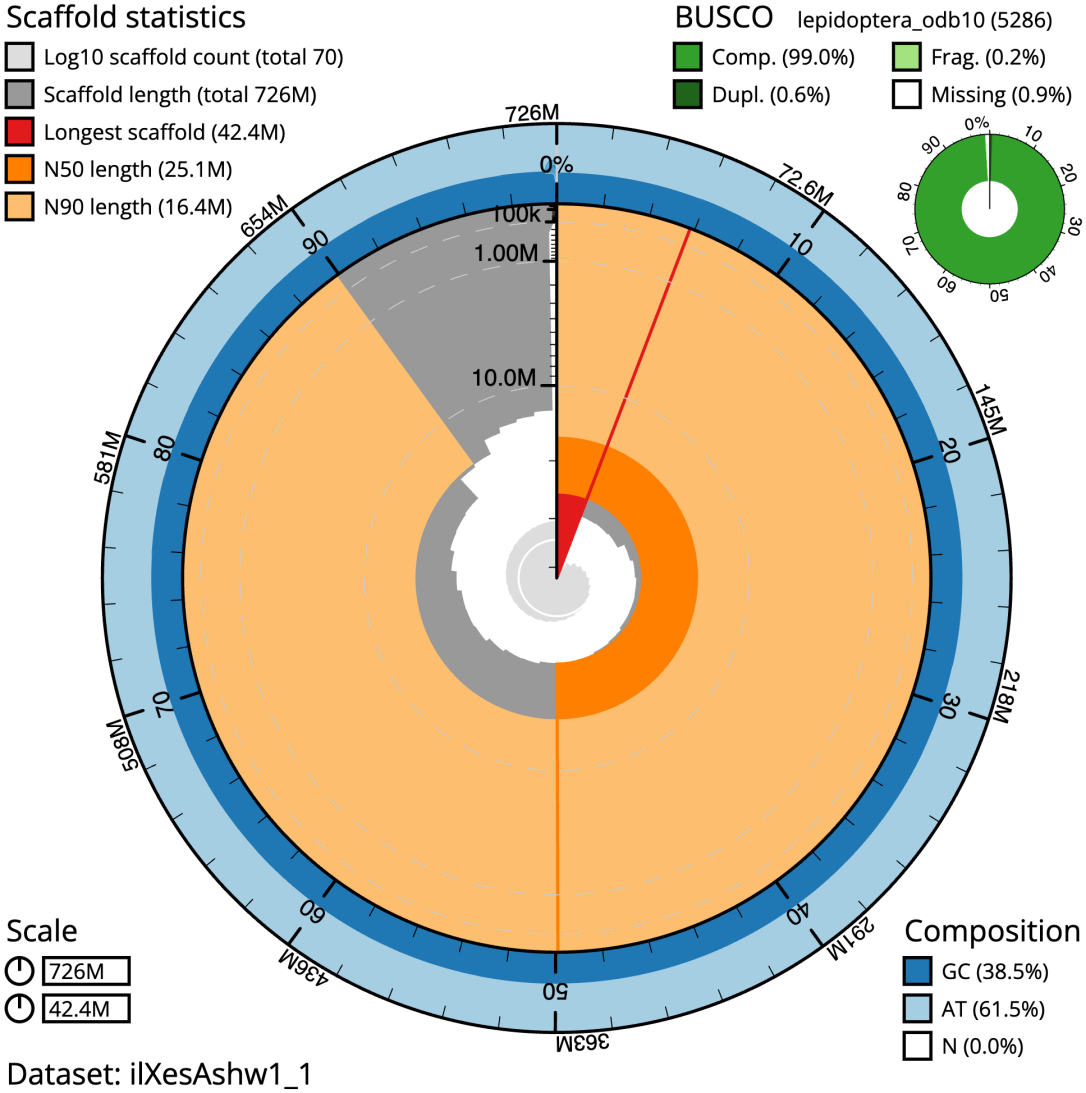
Genome assembly of *Xestia ashworthii*, ilXesAshw1.1: metrics. The BlobToolKit Snailplot shows N50 metrics and BUSCO gene completeness. The main plot is divided into 1,000 size-ordered bins around the circumference with each bin representing 0.1% of the 726,328,142 bp assembly. The distribution of scaffold lengths is shown in dark grey with the plot radius scaled to the longest scaffold present in the assembly (42,390,235 bp, shown in red). Orange and pale-orange arcs show the N50 and N90 scaffold lengths (25,131,726 and 16,416,265 bp), respectively. The pale grey spiral shows the cumulative scaffold count on a log scale with white scale lines showing successive orders of magnitude. The blue and pale-blue area around the outside of the plot shows the distribution of GC, AT and N percentages in the same bins as the inner plot. A summary of complete, fragmented, duplicated and missing BUSCO genes in the lepidoptera_odb10 set is shown in the top right. An interactive version of this figure is available at https://blobtoolkit.genomehubs.org/view/Xestia%20ashworthii/dataset/ilXesAshw1_1/snail.

**Figure 3.**
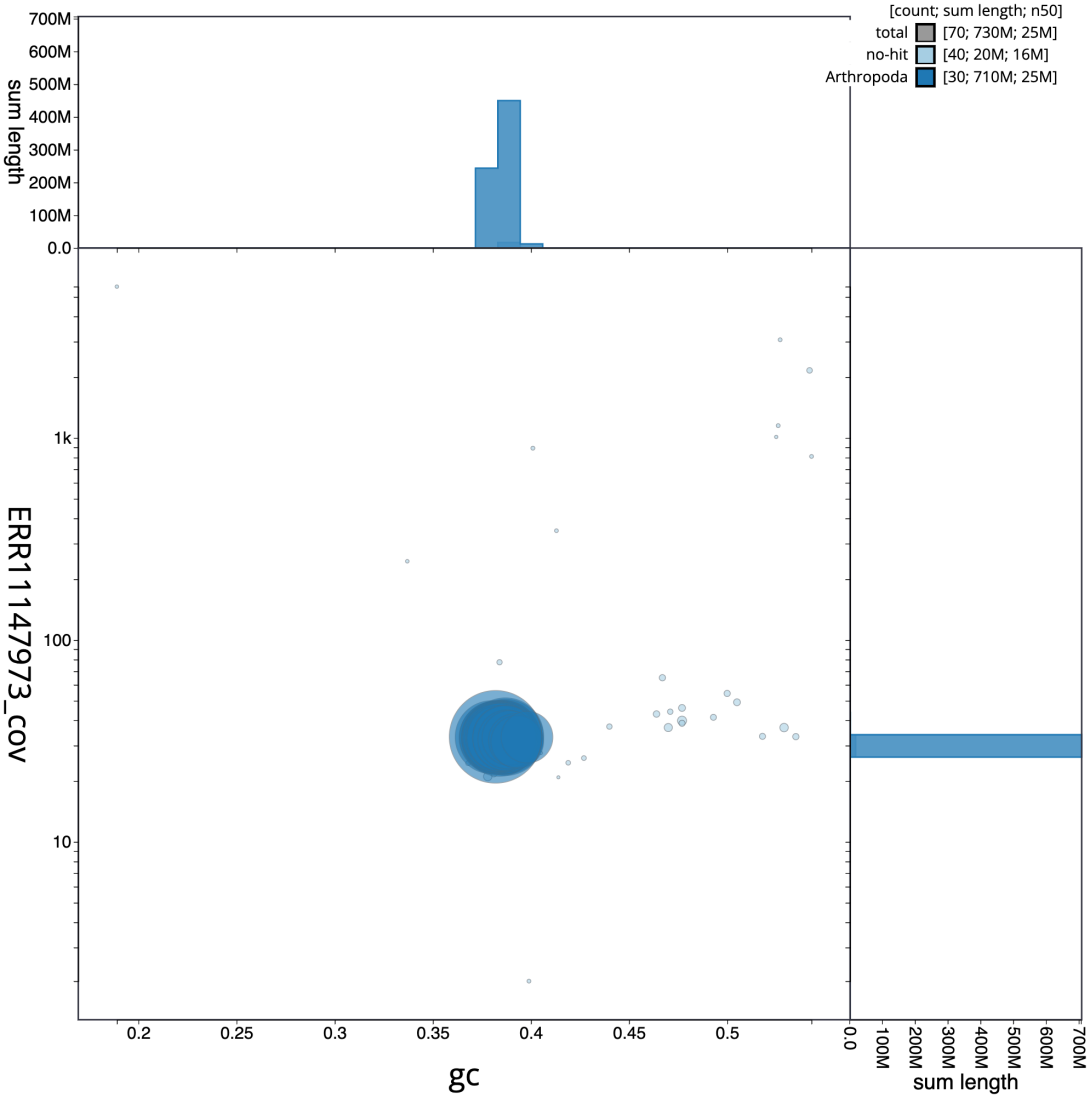
Genome assembly of *Xestia ashworthii*, ilXesAshw1.1: BlobToolKit GC-coverage plot. Scaffolds are coloured by phylum. Circles are sized in proportion to scaffold length. Histograms show the distribution of scaffold length sum along each axis. An interactive version of this figure is available at https://blobtoolkit.genomehubs.org/view/Xestia%20ashworthii/dataset/ilXesAshw1_1/blob.

**Figure 4.**
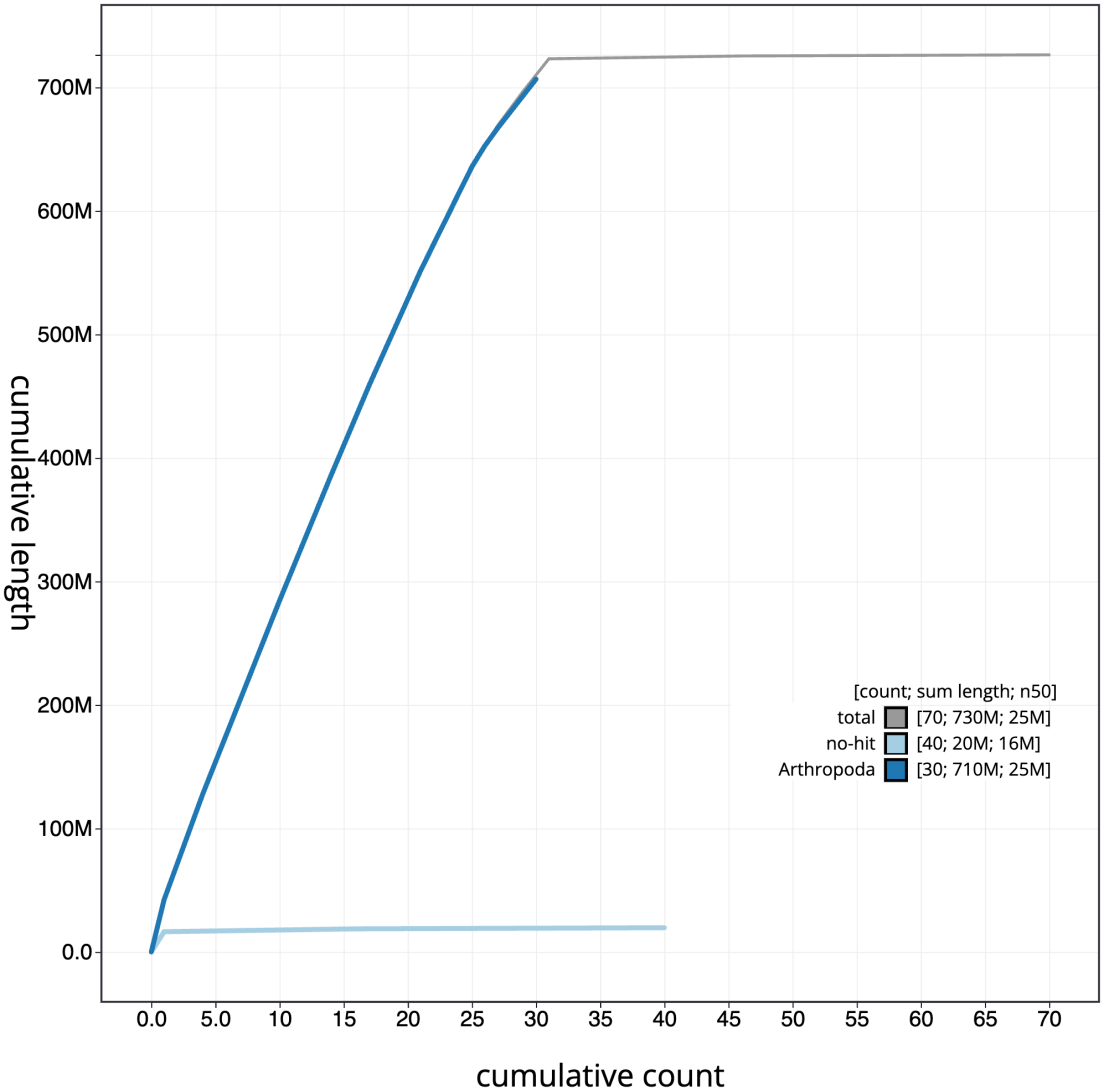
Genome assembly of *Xestia ashworthii*, ilXesAshw1.1: BlobToolKit cumulative sequence plot. The grey line shows cumulative length for all scaffolds. Coloured lines show cumulative lengths of scaffolds assigned to each phylum using the buscogenes taxrule. An interactive version of this figure is available at https://blobtoolkit.genomehubs.org/view/Xestia%20ashworthii/dataset/ilXesAshw1_1/cumulative.

**Figure 5.**
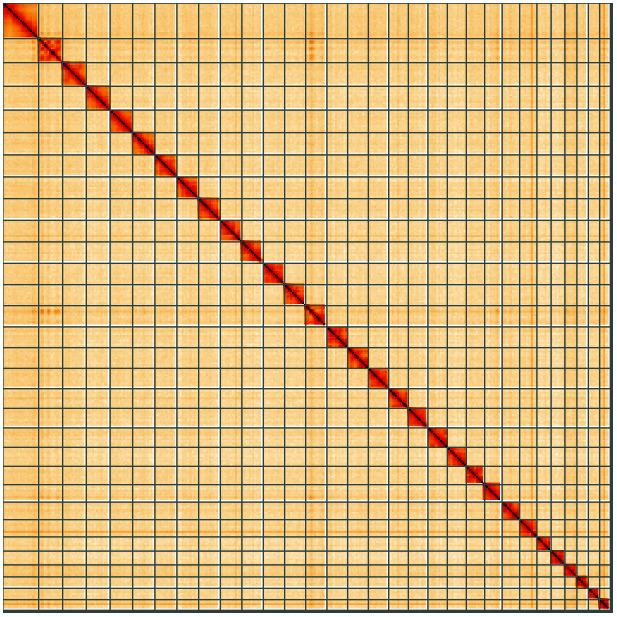
Genome assembly of *Xestia ashworthii*, ilXesAshw1.1: Hi-C contact map of the ilXesAshw1.1 assembly, visualised using HiGlass. Chromosomes are shown in order of size from left to right and top to bottom. An interactive version of this figure may be viewed at https://genome-note-higlass.tol.sanger.ac.uk/l/?d=LKUwmbHETYaIrDZ4kLlArw.

The final assembly has a total length of 726.3 Mb in 69 sequence scaffolds with a scaffold N50 of 25.1 Mb ( Table 1). The snailplot in Figure 2 provides a summary of the assembly statistics, while the distribution of assembly scaffolds on GC proportion and coverage is shown in Figure 3. The cumulative assembly plot in Figure 4 shows curves for subsets of scaffolds assigned to different phyla. Most (99.55%) of the assembly sequence was assigned to 31 chromosomal-level scaffolds, representing 30 autosomes and the Z sex chromosome. Chromosome-scale scaffolds confirmed by the Hi-C data are named in order of size ( Figure 5; Table 2). While not fully phased, the assembly deposited is of one haplotype. Contigs corresponding to the second haplotype have also been deposited. The mitochondrial genome was also assembled and can be found as a contig within the multifasta file of the genome submission.

**Table 1.** Genome data for *Xestia ashworthii*, ilXesAshw1.1.

Project accession data		
Assembly identifier	ilXesAshw1.1	
Species	*Xestia ashworthii*	
Specimen	ilXesAshw1	
NCBI taxonomy ID	988043	
BioProject	PRJEB60829	
BioSample ID	SAMEA111458702	
Isolate information	ilXesAshw1, male: head and thorax (DNA sequencing and Hi-C data), abdomen (RNA sequencing)	
Assembly metrics *		*Benchmark*
Consensus quality (QV)	64	*≥ 50*
*k*-mer completeness	100%	*≥ 95%*
BUSCO **	C:99.0%[S:98.4%,D:0.6%], F:0.2%,M:0.9%,n:5,286	*C ≥ 95%*
Percentage of assembly mapped to chromosomes	99.55%	*≥ 95%*
Sex chromosomes	Z chromosome	*localised homologous pairs*
Organelles	Mitochondrial genome assembled	*complete single alleles*
Raw data accessions		
PacificBiosciences SEQUEL II	ERR11147973	
Hi-C Illumina	ERR11156558	
PolyA RNA-Seq Illumina	ERR12035184	
Genome assembly		
Assembly accession	GCA_950022955.1	
*Accession of alternate haplotype*	GCA_950022645.1	
Span (Mb)	726.3	
Number of contigs	191	
Contig N50 length (Mb)	8.0	
Number of scaffolds	69	
Scaffold N50 length (Mb)	25.1	
Longest scaffold (Mb)	42.4	

* Assembly metric benchmarks are adapted from column VGP-2020 of “Table 1: Proposed standards and metrics for defining genome assembly quality” from ( Rhie *et al.*, 2021). ** BUSCO scores based on the lepidoptera_odb10 BUSCO set using v5.3.2. C = complete [S = single copy, D = duplicated], F = fragmented, M = missing, n = number of orthologues in comparison. A full set of BUSCO scores is available at https://blobtoolkit.genomehubs.org/view/Xestia%20ashworthii/dataset/ilXesAshw1_1/busco.

**Table 2.** Chromosomal pseudomolecules in the genome assembly of *Xestia ashworthii*, ilXesAshw1.

INSDC accession	Chromosome	Length (Mb)	GC%
OX465548.1	1	28.54	38.5
OX465549.1	2	28.41	38.5
OX465550.1	3	28.21	38.5
OX465551.1	4	26.89	38.5
OX465552.1	5	26.36	38.5
OX465553.1	6	26.11	38.5
OX465554.1	7	26.01	38.0
OX465555.1	8	25.95	38.0
OX465556.1	9	25.64	38.5
OX465557.1	10	25.46	38.0
OX465558.1	11	25.28	38.5
OX465559.1	12	25.14	38.5
OX465560.1	13	25.13	38.5
OX465561.1	14	24.75	38.5
OX465562.1	15	24.67	38.5
OX465563.1	16	24.2	38.5
OX465564.1	17	23.49	38.5
OX465565.1	18	23.31	38.5
OX465566.1	19	23.26	38.5
OX465567.1	20	22.55	38.5
OX465568.1	21	21.65	39.0
OX465569.1	22	20.93	38.5
OX465570.1	23	20.82	38.5
OX465571.1	24	20.65	38.5
OX465572.1	25	16.8	39.0
OX465573.1	26	16.42	38.5
OX465574.1	27	14.34	39.0
OX465575.1	28	13.66	39.0
OX465576.1	29	13.3	39.5
OX465577.1	30	12.81	40.0
OX465547.1	Z	42.39	38.0
OX465578.1	MT	0.02	19.0

The estimated Quality Value (QV) of the final assembly is 64 with *k*-mer completeness of 100%, and the assembly has a BUSCO v5.3.2 completeness of 99.0% (single = 98.4%, duplicated = 0.6%), using the lepidoptera_odb10 reference set ( *n* = 5,286).

Metadata for specimens, barcode results, spectra estimates, sequencing runs, contaminants and pre-curation assembly statistics are given at https://links.tol.sanger.ac.uk/species/988043.

## Methods

### Sample acquisition and nucleic acid extraction

A male *Xestia ashworthii* (specimen ID NHMUK014425722, ToLID ilXesAshw1) was collected from Llanfihangel-y-Pennant, Wales, UK (latitude 52.67, longitude –3.96) on 2021-08-01, using a light trap. The specimen was collected and identified by David Lees (Natural History Museum) and preserved by dry freezing at –80 °C.

High molecular weight (HMW) DNA was extracted at the Tree of Life laboratory, Wellcome Sanger Institute (WSI), following a sequence of core procedures: sample preparation; sample homogenisation; HMW DNA extraction; DNA fragmentation; and DNA clean-up. The ilXesAshw1 sample was weighed and dissected on dry ice (as per the protocol https://dx.doi.org/10.17504/protocols.io.x54v9prmqg3e/v1). The head and thorax of the ilXesAshw1 sample was homogenised using a Nippi Powermasher fitted with a BioMasher pestle, following the protocol at https://dx.doi.org/10.17504/protocols.io.5qpvo3r19v4o/v1. DNA was extracted by means of the HMW DNA Extraction: Automated MagAttract protocol ( https://dx.doi.org/10.17504/protocols.io.kxygx3y4dg8j/v1). HMW DNA was sheared into an average fragment size of 12–20 kb in a Megaruptor 3 system with speed setting 30, following the HMW DNA Fragmentation: Diagenode Megaruptor ^®^3 for PacBio HiFi protocol ( https://dx.doi.org/10.17504/protocols.io.8epv5x2zjg1b/v1). Sheared DNA was purified using solid-phase reversible immobilisation (SPRI) (protocol at https://dx.doi.org/10.17504/protocols.io.kxygx3y1dg8j/v1). In brief, the method employs a 1.8X ratio of AMPure PB beads to sample to eliminate shorter fragments and concentrate the DNA. The concentration of the sheared and purified DNA was assessed using a Nanodrop spectrophotometer and Qubit Fluorometer and Qubit dsDNA High Sensitivity Assay kit. Fragment size distribution was evaluated by running the sample on the FemtoPulse system.

RNA was extracted from abdomen tissue of ilXesAshw1 using the Automated MagMax™ *mir*Vana protocol ( https://dx.doi.org/10.17504/protocols.io.6qpvr36n3vmk/v1). The RNA concentration was assessed using a Nanodrop spectrophotometer and Qubit Fluorometer using the Qubit RNA Broad-Range (BR) Assay kit. Analysis of the integrity of the RNA was done using the Agilent RNA 6000 Pico Kit and Eukaryotic Total RNA assay.

All wet lab protocols developed by the Tree of Life laboratory are publicly available on protocols.io: https://dx.doi.org/10.17504/protocols.io.8epv5xxy6g1b/v1.

### Sequencing

Pacific Biosciences HiFi circular consensus DNA sequencing libraries were constructed according to the manufacturers’ instructions. Poly(A) RNA-Seq libraries were constructed using the NEB Ultra II RNA Library Prep kit. DNA and RNA sequencing was performed by the Scientific Operations core at the WSI on Pacific Biosciences SEQUEL II (HiFi) and Illumina NovaSeq 6000 (RNA-Seq) instruments. Hi-C data were also generated from remaining head and thorax tissue of ilXesAshw1 using the Arima2 kit and sequenced on the Illumina NovaSeq 6000 instrument.

### Genome assembly, curation and evaluation

Assembly was carried out with Hifiasm ( Cheng *et al.*, 2021) and haplotypic duplication was identified and removed with purge_dups ( Guan *et al.*, 2020). The assembly was then scaffolded with Hi-C data ( Rao *et al.*, 2014) using YaHS ( Zhou *et al.*, 2023). The assembly was checked for contamination and corrected as described previously ( Howe *et al.*, 2021). Manual curation was performed using HiGlass ( Kerpedjiev *et al.*, 2018) and Pretext ( Harry, 2022). The mitochondrial genome was assembled using MitoHiFi ( Uliano-Silva *et al.*, 2023), which runs MitoFinder ( Allio *et al.*, 2020) or MITOS ( Bernt *et al.*, 2013) and uses these annotations to select the final mitochondrial contig and to ensure the general quality of the sequence.

A Hi-C map for the final assembly was produced using bwa-mem2 ( Vasimuddin *et al.*, 2019) in the Cooler file format ( Abdennur & Mirny, 2020). To assess the assembly metrics, the *k*-mer completeness and QV consensus quality values were calculated in Merqury ( Rhie *et al.*, 2020). This work was done using Nextflow ( Di Tommaso *et al.*, 2017) DSL2 pipelines “sanger-tol/readmapping” ( Surana *et al.*, 2023a) and “sanger-tol/genomenote” ( Surana *et al.*, 2023b). The genome was analysed within the BlobToolKit environment ( Challis *et al.*, 2020) and BUSCO scores ( Manni *et al.*, 2021; Simão *et al.*, 2015) were calculated.

Table 3 contains a list of relevant software tool versions and sources.

**Table 3.** Software tools: versions and sources.

Software tool	Version	Source
BlobToolKit	4.2.1	https://github.com/blobtoolkit/blobtoolkit
BUSCO	5.3.2	https://gitlab.com/ezlab/busco
Hifiasm	0.16.1-r375	https://github.com/chhylp123/hifiasm
HiGlass	1.11.6	https://github.com/higlass/higlass
Merqury	MerquryFK	https://github.com/thegenemyers/MERQURY.FK
MitoHiFi	3	https://github.com/marcelauliano/MitoHiFi
PretextView	0.2	https://github.com/wtsi-hpag/PretextView
purge_dups	1.2.5	https://github.com/dfguan/purge_dups
sanger-tol/genomenote	v1.0	https://github.com/sanger-tol/genomenote
sanger-tol/readmapping	1.1.0	https://github.com/sanger-tol/readmapping/tree/1.1.0
YaHS	1.2a.2	https://github.com/c-zhou/yahs

### Wellcome Sanger Institute – Legal and Governance

The materials that have contributed to this genome note have been supplied by a Darwin Tree of Life Partner. The submission of materials by a Darwin Tree of Life Partner is subject to the **‘Darwin Tree of Life Project Sampling Code of Practice’**, which can be found in full on the Darwin Tree of Life website here. By agreeing with and signing up to the Sampling Code of Practice, the Darwin Tree of Life Partner agrees they will meet the legal and ethical requirements and standards set out within this document in respect of all samples acquired for, and supplied to, the Darwin Tree of Life Project.

Further, the Wellcome Sanger Institute employs a process whereby due diligence is carried out proportionate to the nature of the materials themselves, and the circumstances under which they have been/are to be collected and provided for use. The purpose of this is to address and mitigate any potential legal and/or ethical implications of receipt and use of the materials as part of the research project, and to ensure that in doing so we align with best practice wherever possible. The overarching areas of consideration are:

•   Ethical review of provenance and sourcing of the material

•   Legality of collection, transfer and use (national and international)

Each transfer of samples is further undertaken according to a Research Collaboration Agreement or Material Transfer Agreement entered into by the Darwin Tree of Life Partner, Genome Research Limited (operating as the Wellcome Sanger Institute), and in some circumstances other Darwin Tree of Life collaborators.

## Data Availability

European Nucleotide Archive: *Xestia ashworthii*. Accession number PRJEB60829; https://identifiers.org/ena.embl/PRJEB60829 ( Wellcome Sanger Institute, 2023). The genome sequence is released openly for reuse. The *Xestia ashworthii* genome sequencing initiative is part of the Darwin Tree of Life (DToL) project. All raw sequence data and the assembly have been deposited in INSDC databases. The genome will be annotated using available RNA-Seq data and presented through the Ensembl pipeline at the European Bioinformatics Institute. Raw data and assembly accession identifiers are reported in Table 1.

## Author information

Members of the Natural History Museum Genome Acquisition Lab are listed here: https://doi.org/10.5281/zenodo.7139035.

Members of the Darwin Tree of Life Barcoding collective are listed here: https://doi.org/10.5281/zenodo.4893703.

Members of the Wellcome Sanger Institute Tree of Life Management, Samples and Laboratory team are listed here: https://doi.org/10.5281/zenodo.10066175.

Members of Wellcome Sanger Institute Scientific Operations: Sequencing Operations are listed here: https://doi.org/10.5281/zenodo.10043364.

Members of the Wellcome Sanger Institute Tree of Life Core Informatics team are listed here: https://doi.org/10.5281/zenodo.10066637.

Members of the Tree of Life Core Informatics collective are listed here: https://doi.org/10.5281/zenodo.5013541.

Members of the Darwin Tree of Life Consortium are listed here: https://doi.org/10.5281/zenodo.4783558.
