# Supplementary material for: The genome sequence of Ashworth’s Rustic, Xestia ashworthii (Doubleday, 1855)
Source: Wellcome Open Res. 2023 Dec 20;8:578. [Version 1] doi: 10.12688/wellcomeopenres.20499.1 (PMC11327654; doi:10.12688/wellcomeopenres.20499.1)
Supplement: Peer review history

# Reviewer report: Reviewer response for version 1

**DOI:** 10.21956/wellcomeopenres.22692.r85023

**Published:** 2024-08-29

**Authors:** Kritika Garg

**Affiliations:** 1 Department of Biology, Ashoka University, Sonipat, Haryana, India

The authors describe the genome of Ashworth's Rustic moth. Details of the genome sequencing and the genome are described well. This genome is an important addition to the growing list of Lepidopteran genomes.

 As RNA sequencing was done, the authors can also provide details of the transcriptome data and use it for predicting the proteome.

Are sufficient details of methods and materials provided to allow replication by others?

Yes

Is the rationale for creating the dataset(s) clearly described?

Yes

Are the datasets clearly presented in a useable and accessible format?

Yes

Are the protocols appropriate and is the work technically sound?

Yes

Reviewer Expertise:

Compartive genomics, population genomics, conservation

I confirm that I have read this submission and believe that I have an appropriate level of expertise to confirm that it is of an acceptable scientific standard, however I have significant reservations, as outlined above.

# Reviewer report: Reviewer response for version 1

**DOI:** 10.21956/wellcomeopenres.22692.r91594

**Published:** 2024-08-15

**Authors:** Sivasankaran Kuppusamy

**Affiliations:** 1 Entomology Research Institute, Loyola College, Chennai, India

**Comments on the manuscript:**

- The first sentence of background can be modified as “Ashworth’s Rustic *Xestia ashworthii *(Doubleday, 1855) is a medium-sized (wingspan 16-20 mm) noctuid moth (Waring *et al.,* 2017)”. “that Henry Doubleday based on a specimen…………………..” can be removed. Not necessary to give the details here.

- The second paragraph can be modified as “ *X. ashworthii* is found only Wales in the UK…………………………..”

- The sentence can be modified as “The species was also observed in Anglesey in 1994 (Waring et al., 2017)”.

- Common name of the species may not be used instead of scientific name. Authors have used common name of the species in many places. The species name can be included in the first sentence of the third paragraph. “In Wales there are relatively few records of *X. ashworthii* annually………………………………..”

- Fifth paragraph second sentence within the parenthesis “182 characterise them as polyphagous”.  Is it necessary to give here? Otherwise, details of the polyphagous can be given in separate sentence elaborately. There is little confusion here.

- In the genome sequence report the first sentence can be modified as “The genome was sequenced from a male *X. ashworthii*……”

- In the Methods, First paragraph first sentence can be rewritten as “The genus name can be given in short form like *X. ashworthii*……………………”

- Protein-coding genes and gene transcripts details aren’t given in the article.
- Authors have used standard laboratory protocol for DNA isolation. Software and techniques were for the annotations and assembly of the sequences. 

Above all, I confirm that the manuscript meets the necessary scientific standard and is suitable for indexing.

Are sufficient details of methods and materials provided to allow replication by others?

Partly

Is the rationale for creating the dataset(s) clearly described?

Yes

Are the datasets clearly presented in a useable and accessible format?

Yes

Are the protocols appropriate and is the work technically sound?

Yes

Reviewer Expertise:

Phylogenetic Analysis of Noctuoidea moths

I confirm that I have read this submission and believe that I have an appropriate level of expertise to confirm that it is of an acceptable scientific standard.

# Reviewer report: Reviewer response for version 1

**DOI:** 10.21956/wellcomeopenres.22692.r91598

**Published:** 2024-08-09

**Authors:** Axel Künstner

**Affiliations:** 1 University of Lübeck, Lübeck, Germany

The authors present the genome assembly of *Xestia ashworthii* (Ashworth’s Rustic), a medium-sized noctuid with ZW sex chromosomes. The manuscript is well written and focuses on the genome assembly of the homogametic sex (male). The reported total length of the assembled genome is 726.3Mb in 69 scaffolds, which were assigned to 30 autosomes and the Z chromosome.

 The methods used are sound, software resources used are well described, and the data is available via the European Nucleotide Archive.

Are sufficient details of methods and materials provided to allow replication by others?

Yes

Is the rationale for creating the dataset(s) clearly described?

Yes

Are the datasets clearly presented in a useable and accessible format?

Yes

Are the protocols appropriate and is the work technically sound?

Yes

Reviewer Expertise:

Cancer genomics, Microbiome, Bioinformatics, Computational Biology

I confirm that I have read this submission and believe that I have an appropriate level of expertise to confirm that it is of an acceptable scientific standard.

# Reviewer report: Reviewer response for version 1

**DOI:** 10.21956/wellcomeopenres.22692.r75003

**Published:** 2024-05-11

**Authors:** Josephine Paris

**Affiliations:** 1 Department of Health, Life and Environmental Sciences, University of L'Aquila, Coppito, Italy

In this report, the assembled genome of *Xestia ashworthii* is presented. The Introduction provides a nice and very detailed background on the species and its distribution. I only stumbled upon one part, which reads, "Heath Bedstraw ( *Galium saxatile*) and heathers seem to be the most important foodplants ( Bretherton *et al.*, 1983: 182 characterise them as polyphagous" It would be nice to know what the 182 refers to, as currently it is unclear to me. The assembly was performed to a good standard using Pacbio Hifi data and scaffolded with Hi-C data. Assembly contiguity and completeness are well demonstrated. It would be nice to include the amount (in Gb) of Pacbio Hifi (not only the coverage) and Hi-C data that was generated. The datasets are clearly available via the links provided.

Are sufficient details of methods and materials provided to allow replication by others?

Yes

Is the rationale for creating the dataset(s) clearly described?

Yes

Are the datasets clearly presented in a useable and accessible format?

Yes

Are the protocols appropriate and is the work technically sound?

Yes

Reviewer Expertise:

Population genomics, genome assembly and annotation

I confirm that I have read this submission and believe that I have an appropriate level of expertise to confirm that it is of an acceptable scientific standard, however I have significant reservations, as outlined above.
